# Study on the Pauropoda from Tibet, China. Part I. The genera *Decapauropus* and *Hemipauropus* (Myriapoda)

**DOI:** 10.3897/zookeys.754.24210

**Published:** 2018-04-30

**Authors:** Chang-Yuan Qian, Yun Bu, Yan Dong, Yun-Xia Luan

**Affiliations:** 1 Key Laboratory of Insect Developmental and Evolutionary Biology, Institute of Plant Physiology and Ecology, Chinese Academy of Sciences, Shanghai 200032, China; 2 Natural History Research Center, Shanghai Natural History Museum, Shanghai Science & Technology Museum, Shanghai 200041, China; 3 College of Biology and Food Engineering, Chuzhou University, Chuzhou 239000, China

**Keywords:** diversity, Motuo County, new record, new species, Pauropodidae, taxonomy

## Abstract

Three new species of family Pauropodidae: *Decapauropus biconjugarus* Qian & Bu, **sp. n.**, *D. tibeticus* Qian & Bu, **sp. n.** and *Hemipauropus quadrangulus* Qian & Bu, **sp. n.** are described and illustrated from southeastern Tibet, China. The genus *Hemipauropus* is recorded for the first time from China. This is the second report of pauropods from Tibet.

## Introduction

To date, there is only one species of Pauropoda reported in Tibet (Zhang and Chen 1988). Since only a single specimen was obtained and was tentatively identified as *Sphaeropauropus* sp. of the family Sphaeropauropodidae, Silvestri, 1930. For the last thirty years, this remained the only record of Pauropoda from Tibet. In November 2015, a short expedition to Motuo and Bomi counties, southeastern Tibet of China was carried out. In total, 50 specimens of Pauropoda were obtained during the expedition. In the present study, we describe and illustrate three new species of the family Pauropodidae Lubbock, 1867, including one species belonging to the genus *Hemipauropus*, which is recorded for the first time from China. The other two species belong to the genus *Decapauropus*. This is the second report of pauropods from the territory of Tibet.

## Materials and methods

Sampling was made in three areas of southeastern Tibet in 2015: Dexing town, Motuo County; Beibeng town, Motuo County, and Songzong town, Bomi County. Pauropods were collected by means of Tullgren’s funnel. Specimens were sorted under a stereo dissection microscope and preserved in 80% alcohol. Each specimen was mounted with Hoyer’s solution and identified under a phase contrast microscope (Nikon ECLIPSE N*i*, objective lens 100X / 1.30 Oil, ∞/ 0.17 WD 0.20 (0.16)). All specimens were deposited in the collection maintained by the Shanghai Natural History Museum (**SNHM**) and the Shanghai Entomological Museum (**SEM**).

Abbreviations used in the descriptions:

**Head. *a*_1_** – a submedian pair of setae on tergal side of head, ***a*_2_** – an intermediate pair of setae on tergal side of head, ***a*_3_** – a sublateral pair of setae on tergal side of head, ***a*_4_** – a posterolateral pair of setae on head.

**Antenna. *bs*** – base segment of antennal flagellum, ***F*_1_** – flagellum of tergal antennal branch; ***F*_2_** – anterior flagellum of sternal antennal branch, ***F*_3_** – posterior flagellum of sternal antennal branch, ***g*** – globulus of sternal antennal branch, ***p*** – a tergal seta on fourth antennal segment, ***p***’ – an anterior seta on fourth antennal segment, ***p***” – a sternal seta on fourth antennal segment, ***q*** – a seta on sternal side of sternal antennal branch, ***r*** – a posterior seta on fourth antennal segment, ***s*** – sternal antennal branch, ***t*** – tergal antennal branch.

**Trunk. *T*_1–5_** – first to fifth pair of bothriotricha on tergites.

**Pygidial tergum. *a*_1_** – (sub) median pair of setae, ***a*_2_** – intermediate pair of setae, ***a*_3_** – sublateral pair of setae, ***st*** – styli.

**Pygidial sternum. *b*_1_** – posterior pair of setae, ***b*_2_** – lateral pair of setae, ***b*_3_** – anterior pair of setae.

Measurements are provided as length of body in mm; the range of variation in adult paratypes is given in brackets. Absolute lengths of all other body parts are given in μm. Otherwise, the text refers relative lengths.

## Results

### Taxonomy

#### Family Pauropodidae

##### Genus *Decapauropus* Remy, 1931

###### Decapauropus biconjugarus

Taxon classificationAnimaliaORDOFAMILIA

Qian & Bu sp. n.

http://zoobank.org/2E2C1271-92AD-4001-8192-9A652779E81D

Figs 1 , 2

####### Material examined.

Holotype, adult with 9 pairs of legs, female (slide no. XZ-PA2015025) (SNHM), China, Tibet, Motuo county, Dexing town, extracted from soil samples in a broad-leaved forest, alt. 1100 m, 29°40'N, 95°26'E, 3-XI-2015, coll. Y. Bu & G. Yang. Paratypes, 2 adults, with 9 pairs of legs, females (slides no. XZ-PA2015019 (SNHM), XZ-PA2015026 (SEM)), 2 subadults, with 8 pairs of legs (slides no. XZ-PA2015027 (SNHM), XZ-PA2015031 (SEM)), same data as holotype.

####### Etymology.

From the Latin *biconjugarus* referring to the anal plate with two pairs of clavate appendages.

####### Diagnosis.

*Decapauropus biconjugarus* sp. n. is distinguished from the other species in the genus by the shape of the anal plate: subquadrate, with obvious U-shape and concave lateral margins; distal part with 4 posteriorly directed clavate appendages, dorsal ones thickest, straight, annulate, those protruding from sternal side shorter and thinner, straight, glabrous. Posterior part of the pygidial sternum evenly rounded.

####### Description.

Holotype length 0.6 mm (Fig. 2A).

**Figure 1.**
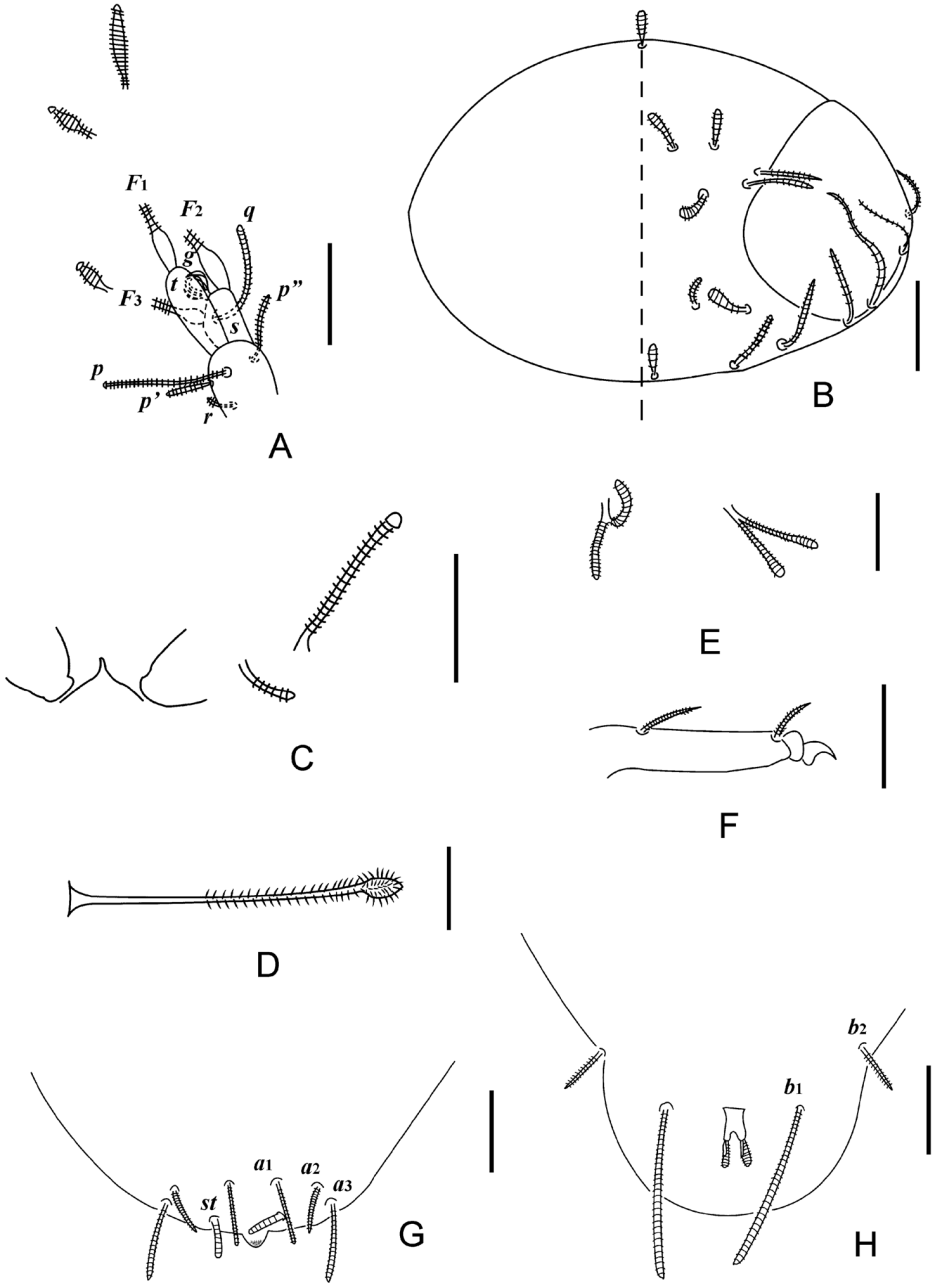
*Decapauropus biconjugarus* sp. n. (holotype) **A** Left antenna, tergal view **B** Head, median and right part, dorsal view **C** Collum segment, median and left part, sternal view **D** *T*_3_ **E** Setae on coxa (left) and trochanter (right) of leg IX **F** Tarsus of leg IX **G** tergum of pygidum **H** sternum of pygidum. Scale bars: 20 μm.

**Figure 2.**
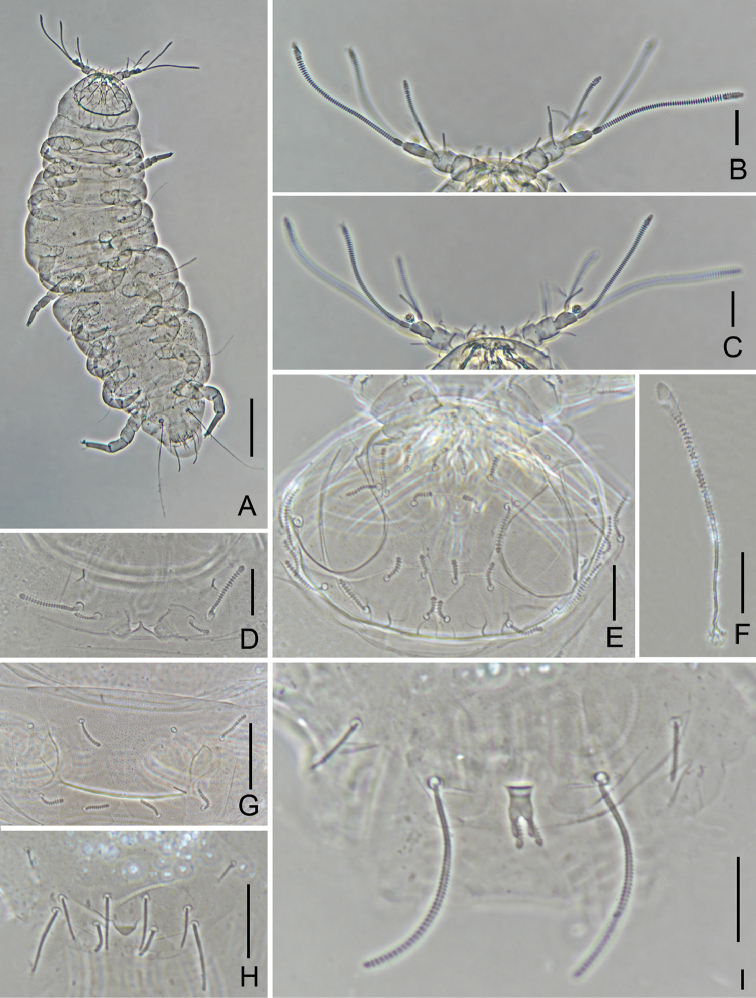
*Decapauropus biconjugarus* sp. n. (holotype) **A** Habitus **B** Antenna, tergal view **C** Antenna, sternal view **D** Collum segment, sternal view **E** Head, dorsal view **F** *T*_3_ **G** Tergite I **H** Tergum of pygidum **I** Sternum of pygidum. Scale bars: 100 μm (**A**), 20 μm (**B–I**).

*Head* (Figs 1B, 2E). Dorsal head setae short to moderately long, clavate, lateral ones cylindrical. Relative lengths of setae, 1^st^ row: *a*_1_ = 10, *a*_2_ = 8 (10); 2^nd^ row: *a*_1_ = (9) 10, *a*_2_ = 18 (19) *a*_3_ = 14; 3^rd^ row: *a*_1_ = 8 (10), *a*_2_ = (10) 12; 4^th^ row: *a*_1_ = 8, *a*_2_ = (16) 18, *a*_3_ = (18) 20, *a*_4_ = (30) 32; lateral group setae *l*_1_ =26 (27), *l*_2_ = 22 (25) *l*_3_ = 20 (30); the ratio *a*_1_/*a*_1_–*a*_1_ in 1^st^ row 0.8, 2^nd^ row 0.4, 3^rd^ row 0.4 and 4^th^ row 0.4. Temporal organs oval in dorsal view, their length 1.4 times as long as their shortest distance apart. Head cuticle glabrous.

*Antennae* (Figs 1A, 2B, 2C). Antennal segment 4 with four cylindrical setae; relative lengths of setae: *p* = 100, *p*’ = (39.9) 41.4, *p*’’ = 62.5 (63.8), *r* = (26) 27.6; tergal seta *p* (1.2) 1.3 times as long as tergal branch *t.* The latter cylindrical, 2.2 (2.3) times as long as its greatest diameter and (1.6) 1.7 times as long as sternal branch *s*, which itself is 2.3 times as long as its greatest diameter. Seta *q* cylindrical, blunt, 1.3 times as long as *s. F*_3_ very thin with small base segment. Relative lengths of flagella (base segments included) and base segments: *F*_1_ = 100, *bs*_1_ = 6; *F*_2_ = 44.4, *bs*_2_ = 4; *F*_3_ =83.3, *bs*_3_ = 5. *F*_1_ 4.5 times as long as *t, F*_2_ and *F*_3_ 2.7 and 5.0 times as long as *s* respectively. Distal calyces small; *F*_1_ and *F*_2_ with fusiform flagella axes just below calyx. Distal calyces spherical; distal part of flagella axes fusiform. Globulus *g* 1.75 times as long as wide; about 12 bracts, capsule subspherical; width of *g* 0.67 of the greatest diameter of *t.* Antennae nearly glabrous.

*Trunk.* Setae on collum subcylindrical, striate, and appearing simple. Sublateral setae length 27 μm, 2.5 times as long as submedian setae (Figs 1C, 2D); sternite process triangular, pointed; appendages narrowing distally and with flat caps (Figs 1C, 2D). Setae on tergites thin, cylindrical; 4 + 4 setae on tergite I (Fig. 2 G), 6 + 6 on II–IV, 6 + 4 on V, 4 + 2 on VI. Tergites glabrous.

*Bothriotricha*. Relative lengths: *T*_1_ = 100, *T*_2_ = 126.7, *T*_3_ = 106.7, *T*_4_ =128.0, *T*_5_ = 206.7. Axes simple, straight, in all but *T*_3_ very thin; axes of *T*_3_ thickened in distal half (Figs 1D, 2F). Pubescent hairs simple, short, thin, strongest on distal half of *T*_3_.

*Legs.* Setae on coxa and trochanter of leg IX length 20 and 23 μm respectively, furcate with subcylindrical, annulate, blunt branches (Fig. 1E). Tarsus of leg IX long, 45 μm, tapering, 4.1 times as long as its greatest diameter (Fig. 1F). Proximal seta long, 14 μm, tapering, striate; distal seta 11 μm, tapering, striate; their lengths 0.35 and 0.30 of the tarsal length, respectively. Cuticle of tarsus glabrous.

*Pygidum. Tergum* (Figs 1G, 2H). Posterior margin evenly rounded but with small median triangular lobe between *a*_1_ and *st*, the lobe granulated distally. Relative lengths of setae: *a*_1_ = 100, *a*_2_ = 82.4, *a*_3_ = 117.6, *st* = 58.8. All setae subcylindrical, blunt, striate; *st* convergent; Distance *a*_1_–*a*_1_ 0.64 of length of *a*_1_; distance *a*_1_–*a*_2_ 3.3 times as long as *a*_2_–*a*_3_; distance *st*–*st* 1.5 times as long as *st* and 1.4 times as long as distance *a*_1_–*a*_1_. *Sternum* (Figs 1H, 2I). Posterior margin evenly rounded and smooth between *b*_1_. Relative lengths of setae (*a*_1_ =100): *b*_1_ = 235.3, *b*_2_ = 82.4. All setae subcylindrical, blunt, striate. Distance *b*_1_–*b*_1_ 0.7 of length of *b*_1_; distance *b*_1_–*b*_2_ 1.1 times as long as *b*_2_.

*Anal plate* subquadrate, with obvious U shape concave lateral margins; distal part with four posteriorly directed clavate appendages, tergal ones thickest, straight, annulate, those protruding from sternal side shorter and thinner, straight, glabrous. Tergal and sternal appendages 0.9 and 0.5 times as long as plate respectively. Plate and sternum glabrous.

####### Remarks.

This new species seems to be a very close relative of *D. bedosae* Scheller from north-western Thailand (Scheller 1995) and *D. cibodasensis* Scheller from Singapore (Scheller 2007). They can be distinguished by the shape of the posterior part of the pygidial sternum (margin evenly rounded in the new species vs. straight in *D. cibodasensis*; with broad indentation in *D. bedosae*) and by the shape of the anal plate (plate short with medium appendages in *D. biconjugarus*; plate short with long appendages in *D. cibodasensis*; plate longer with sort appendages, especially the sternal ones in *D. bedosae*).

###### Decapauropus tibeticus

Taxon classificationAnimaliaORDOFAMILIA

Qian & Bu sp. n.

http://zoobank.org/353D5D52-EE05-4DA6-A063-77441C7E6C61

Figs 3 , 4

####### Material examined.

Holotype, adult with nine pairs of legs, female (slide no. XZ-PA2015007) (SNHM), China, Tibet, Linzhi City, Bomi county, Songzong town, extracted from soil samples in a broad-leaved forest, Alt. 3000 m, 29°76'N, 95°96'E, 7-XI-2015, coll. Y. Bu & G. Yang. Paratype, adult with 9 pairs of legs, female (slide no. XZ-PA2015009) (SNHM), same data as holotype.

####### Etymology.

The species is named after Tibet.

####### Diagnosis.

*Decapauropus tibeticus* sp. n. is distinguished from the other species in the genus by the shape of the anal plate bearing comma shaped appendages with pubescence. This in combination with *st* expanded and annulate distally is a very peculiar character for members of this genus.

####### Description.

Holotype length 0.72 mm (Fig. 4A), paratype length 0.77 mm.

**Figure 3.**
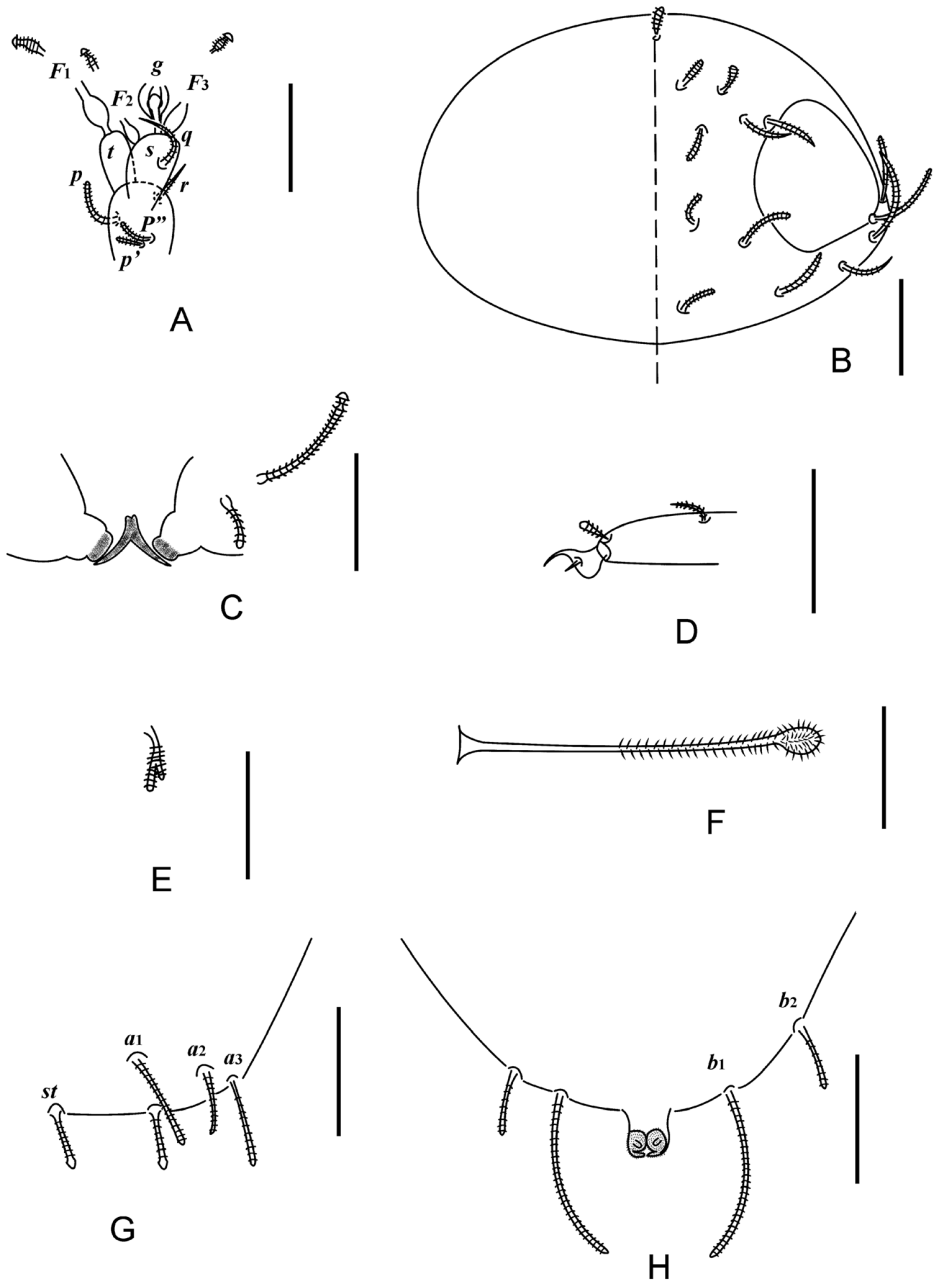
*Decapauropus tibeticus* sp. n. (holotype) **A** Right antenna, sternal view **B** Head, median and right part, dorsal view **C** Collum segment, median and left part, sternal view **D** Tarsus of leg IX **E** Setae on trochanter of leg IX **F** T3 **G** Tergum of pygidum **H** Sternum of pygidum. Scale bars: 20 μm.

**Figure 4.**
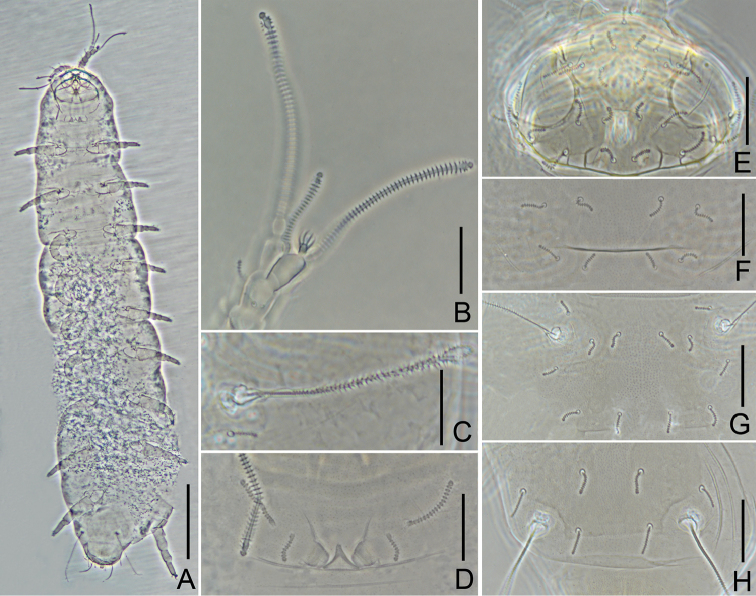
*Decapauropus tibeticus* sp. n. (holotype) **A** Habitus **B** Right antenna, sternal view **C** *T*_3_ **D** Collum segment, sternal view **E** Head, dorsal view **F** Tergite I **G** Tergite II **H** Tergite VI. Scale bars: 100 μm (**A**), 20 μm (**B–H**).

*Head* (Figs 3B, 4E). Dorsal head setae short, blunt, densely annulate. Relative lengths of setae: 1^st^ row: *a*_1_ = 10, *a*_2_ = 12 (8.6); 2^nd^ row: *a*_1_ = 12 (10), *a*_2_ = 22 (14.3), *a*_3_ = 18 (14.3); 3^rd^ row: *a*_1_ = 16 (10), *a*_2_ = 18 (12.9); 4^th^ row: *a*_1_ = 18 (8.6), *a*_2_ = 24 (17.1), *a*_3_ = 26 (12.9), *a*_4_ = 22.9 (?); lateral group setae *l*_1_ = (20) 22.5, *l*_2_ = (17) 20, *l*_3_ = 14 (?). Ratio *a*_1_/*a*_1_‒*a*_1_ in 1^st^ row 0.6, in 2^nd^ row 0.4, in 3^rd^ row 0.3, in 4^th^ row 0.75. Length of temporal organs 1.2 times as long as shortest interdistance. Head cuticle glabrous.

*Antennae* (Figs 3A, 4B). Antennal segment 4 with four cylindrical, annulate setae. Relative lengths of setae: *p* = 100, *p*’ = 46.2 (77.8), *p*” = 53.8 (66.7), *r* = 61.5 (88.9). Tergal seta *p* 1.3 (0.9 of) times as long as tergal branch *t.* The latter slender, cylindrical, 1.7 times as long as greatest diameter and 0.8 of sternal branch *s* which is 2.2 (1.5) times as long as greatest diameter; seta *q* 0.9 of sternal branch *s.* Relative lengths of flagella (base segment included) and base segments: *F*_1_ = 100, *bs*_1_ = 7.9 (8), *F*_2_ = 36.5 (33.3), *bs*_2_ = 13 (12), *F*_3_ = 85.7 (73.3), *bs*_3_ = 9.3 (9.1). *F*_1_ 6.3 (7.5) times as long as *t. F*_2_ and *F*_3_ 1.8 (2.1) and 4.2 (4.6) times as long as *s*, respectively. Globulus *g* 1.8 (1.6) times as long as greatest diameter; width of *g* 0.7 (0.8) of greatest diameter of *t*.

*Trunk.* Setae on collum segment subcylindrical, simple, annulate (Figs 3C, 4D). Sublateral setae length 21 μm, 2.1 times as long as submedian setae (10 μm); sternite process thin, pointed anteriorly, with a little incision; appendages with low caps (Fig. 3C). Process and appendages with particles. Seta on tergites annulate, 4+4 on tergite I (Fig. 4F), 6+6 on II‒IV (Fig. 4G), 6+4 on V, 4+2 on VI (Fig. 4H).

*Bothriotricha*. Relative lengths: *T*_1_ = 100, *T*_2_ = 107.7 (107.1), *T*_3_ = 92.3 (92.9), *T*_4_ = 107.7 (107.1), *T*_5_ = 123.1 (121.4). All but *T*_3_ with very thin, simple straight axes and with short oblique pubescence. Axes of *T*_3_ thickened in distal half. Pubescent hairs simple, short, thin, strongest on distal half of *T*_3_ (Figs 3F, 4C).

*Legs.* Coxa and trochanter of leg IX with furcate setae, lengths 10 and 11 μm respectively, branches subcylindrical, blunt (Fig. 3G). Tarsus of leg IX short, 23 μm (Fig. 3D), somewhat tapering, 2.7 (2.9) times as long as greatest diameter; setae on similar appearance, thin, cylindrical, annulate, length 6‒7 μm, approx. 0.2 of length of tarsus.

*Pygidum. Tergum* (Fig. 3G). Posterior margin of pygidial tergum evenly rounded. Relative lengths of setae: *a*_1_=100, *a*_2_ = 86.7, *a*_3_ = 113.3, *st* = 66.7. All setae but *st* blunt, annulate; *st* subcylindrical, straight, annulate, with a little expanding distally; Distance *a*_1_‒*a*_1_ 0.73 of *a*_1_; distance *a*_1_‒*a*_2_ 2.7 times as long as *a*_2_–*a*_3_; distance *st*–*st* 1.7 times as long as *st* and *st*–*st* 1.3 times as long as distance *a*_1_‒*a*_1_. *Sternum* (Fig. 3H). Posterior margin of sternum evenly rounded. Relative lengths of setae (*a*_1_=100): *b*_1_ = 233.3 (187.5), *b*_2_ = 80 (68.8). All setae cylindrical, annulate. Distance *b*_1_‒*b*_1_ 1.8 times as long as length of *b*_1_; distance *b*_1_‒*b*_2_ 0.16 of length of *b*_2_.

*Anal plate* subsquare, glabrous, width 0.9 of length, posterior margin with two short, comma shaped, pubescent appendages, appendages with a pair of little stubs that are almost half length of plate.

####### Remarks.

The species differs significantly from the other congeners. The comma shaped appendages of the anal plate with a pair of little stubs are characters unknown in other members of the genus.

##### Hemipauropus Silvestri, 1902, new record to China.

**Type species.** *Hemipauropus leptoproctus* Silvestri, 1902

**Diagnosis.** Preanal segment much narrower than other body segments, cuticles of tergites with reticulations, particularly on most anterior and posterior parts; pygidial sternum with one pair of seta, *b*_1_.

**Distribution.** Palaearctic region; Neotropical region; Ethiopian region; Oriental region; Australian region.

###### Hemipauropus quadrangulus

Taxon classificationAnimaliaORDOFAMILIA

Qian & Bu sp. n.

http://zoobank.org/15D1D101-8426-49BC-A74B-AC15355BA09E

Figs 5 , 6

####### Material examined.

Holotype, adult with nine pairs of legs, male (slide no. XZ-PA2015037) (SNHM), China, Tibet, Motuo county, Beibeng town, extracted from the soil samples in a broad-leaved forest, alt. 1500 m, 29°30'N, 95°38'E, 5-XI-2015, coll. Y. Bu & G. Yang.

####### Etymology.

From Latin *quadrangulus* meaning four angles and referring to the shape of the base of the anal plate.

####### Diagnosis.

*Hemipauropus quadrangulus* sp. n. is distinguished from the other species in the genus by the shape of the anal plate, which has a peculiar small Shuriken base and 6+6 setae on tergite IV.

####### Description.

Length. 0.85 mm (Fig. 6A).

**Figure 5.**
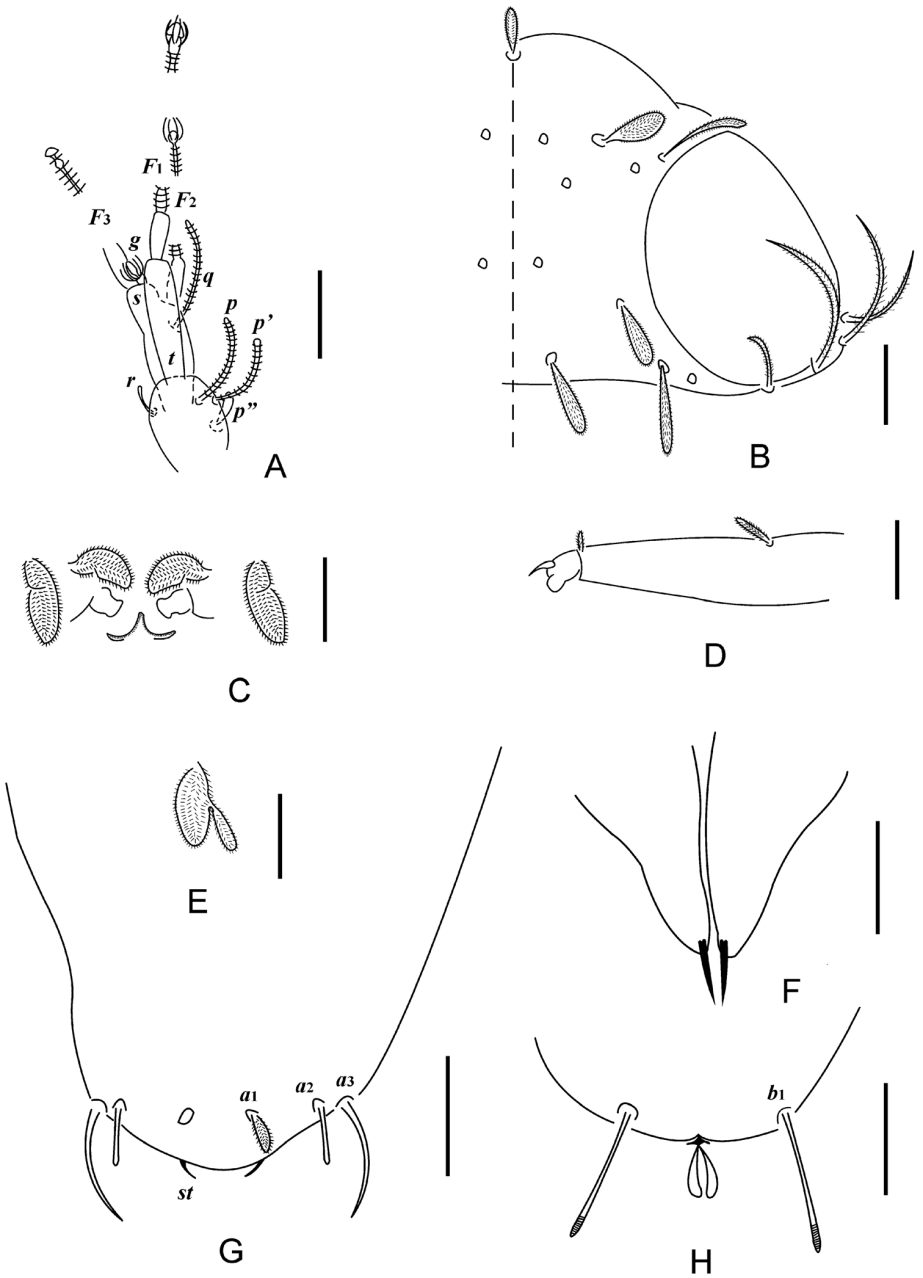
*Hemipauropus quadrangulus* sp. n. (Holotype) **A** Left antenna, tergal view **B** Head, median and right part, dorsal view **C** Collum segment, sternal view **D** Tarsus of leg IX **E** Setae on trochanter of leg IX **F** Genital papillae **G** Tergum of pygidum **H** Sternum of pygidum. Scale bars: 20 μm.

*Head* (Fig. 5B). Tergal setae rather long, leaf-shaped, with short pubescence, lateral setae including *a*3 of 2^nd^ row and *a*4 of 4^th^ row, cylindrical, tapering in distal half, pointed. Relative lengths of setae, 1^st^ row: *a*_1_ = ?, *a*_2_ =10; 2^nd^ row: *a*_1_ = ?, *a*_2_ = ?; *a*_3_ = 15; 3^rd^ row: *a*_1_ = ?, *a*_2_ = 10.7; 4^th^ row: *a*_1_ = 11.4, *a*_2_ = 12.9, *a*_3_ = ?, *a*_4_ = 10; lateral group setae: *l*_1_ = 24.3, *l*_2_ = 25.7, *l*_3_ = 20. Ratio *a*_1_/*a*_1_–*a*_1_ in 1^st^ to 3^rd^ row unknown, in 4^th^ row 0.9. Temporal organs oval in tergal view, length 1.4 of shortest interdistance; pistil absent. Head cuticle with very fine granules, temporal organs glabrous.

**Figure 6.**
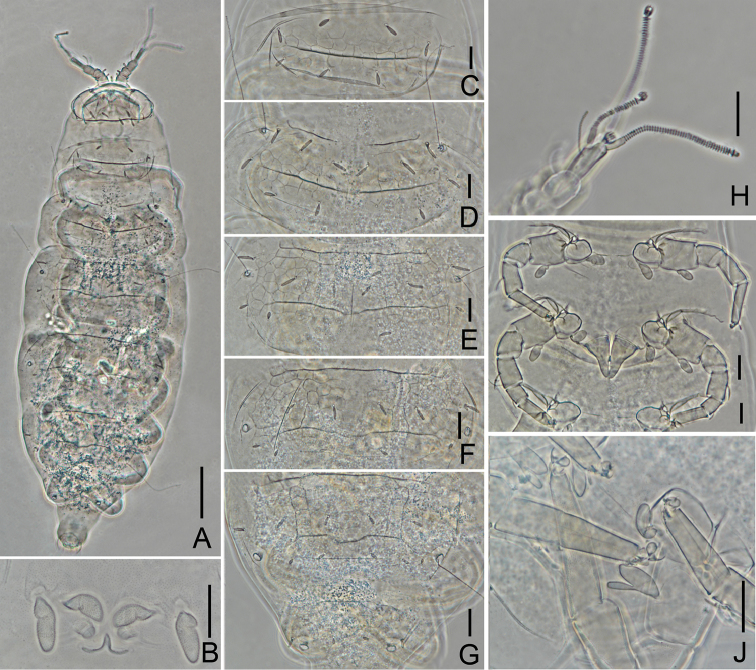
*Hemipauropus quadrangulus* sp. n. (Holotype) **A** Habitus **B** Collum segment, sternal view **C** Tergite I **D** Tergite II **E** Tergite III **F** Tergite IV **G** Tergite V–VI **H** Right antenna, sternal view **I** Legs I–II and genital papillae **J** Leg IX. Scale bars: 100 μm (**A**), 20 μm (**N–J**).

*Antennae* (Figs 5A, 6H). Setae on segments 1–3 folioform. Segment 4 with four setae, *p* and *p*’ subcylidrical, *p*” leaf-shaped, *r* very thin, *p*’ and *p*” striate; relative lengths of setae: *p* = 10, *p*’ = 9.3, *p*” = 3.3, *r* = 4. Tergal branch *t* somewhat fusiform, 3.8 times as long as greatest diameter and 0.95 of the length of sternal branch *s*; that branch 2.9 times as long as greatest diameter; anterodistal corner truncated. Seta *q* 1.3 times as long as seta *p*’ of segment 4, 0.95 of the length of *s.* Relative lengths of flagella (base segments included) and base segments: *F*_1_ = 100, *bs*_1_ = 17.3; *F*_2_ = 46.7, *bs*_2_ = 14.7; *F*_3_ = 101.3, *bs*_3_ = 14.7. *F*_1_ 3.3 times as long as *t, F*_2_ and *F*_3_ 1.2 and 2.5 times as long as *s* respectively. Distal organ of *F*_1_ and *F*_2_ consisting of densely arranged pubescent bracts around sessile capsule, *F*_3_ with flat calyx; flagella axes below distal organs not widened in *F*_1_ and *F*_2_, slightly in *F*_3_. Globulus *g* pyriform, 0.2 of the length of *s*, diameter 0.8 of greatest diameter of *t*; 8–10 bracts; capsule subspherical. Antennae almost glabrous.

*Trunk.* Setae of collum segment broad, phylliform, pubescent, secondary branch rudimentary and inserted just below the middle; sublateral setae length 20 μm, 1.3 times as long as submedian setae; sternite process broad, pointed anteriorly; appendages two-parted with low caps. Process with pubescence and appendages glabrous (Figs 5C, 6B). Tergites indistinctly divided transversally, II–IV with reticular pattern on both sides of the dividing line, only posterior of that line on VI (Fig. 6C–G). Setae cylindrical, 4+4 setae on tergite I (Fig. 6C), 6+6 on II–V (Figs 6 D–G), 4 only on VI (Fig. 6G). Submedian posterior setae on VI 0.2 of inter-distance and 0.4 (–0.5) of the length of pygidial setae *a*_1_. Tergites glabrous.

*Bothriotricha*. Relative lengths: *T*_1_ = 100, *T*_2_ = 104.8, *T*_3_ = ?, *T*_4_=104.8, *T*_5_ = ?. Axes thin, simple, straight, pubescence hairs exceedingly short.

*Genital papillae* (Figs 5F, 6I). Longish, conical with narrowing and extended distal half, 1.6 times as long as wide, seta short, 0.3 of the length of papilla. Coxal seta of leg II as on leg I, length 10 μm (Fig. 6I).

*Legs.* Fairly long. Setae on coxa and trochanter of leg IX furcate, main branch leaf-shaped, secondary branch subcylindrical, blunt, 0.5 of the length of seta (Figs 5E, 6J). Tarsus of leg IX long, broad, 60 μm in length, 4.3 times as long as greatest diameter; proximal seta short, blunt, length 10 μm, with pubescence, distal seta shorter and blunt, length 9 μm, with pubescence. Proximal seta 0.2 of the length of tarsus, 2.5 times as long as distal seta. Cuticle of tarsus almost glabrous (Fig. 5D).

*Pygidium. Tergum* (Fig. 5G). Cuticle glabrous. Posterior margin smooth and round between *st.* Setae of very different lengths, *a*_1_ leaf shaped, pubescent, *a*_2_ subcylindrical, almost straight, *a*_3_ thin, tapering, pointed, *a*_2_ and *a*_3_ glabrous. *st* very short, pointed, converging; relative lengths of setae: *a*_1_ = 10, *a*_2_ = 13.8, *a*_3_ = 31.3, *st* = 6.3. Distance *a*_1_–*a*_1_ 1.6 times as long as *a*_1_; distance *st*–*st* 3.0 times as long as *st* and 1.2 times as long as distance *a*_1_–*a*_1_. *Sternum* (Fig. 5H). Posterior margin with a little indention; *b*_1_ blunt, striate distally. Relative lengths (pygidial *a*_1_ = 10), *b*_1_ = 27.8, 0.8 of their inter-distance.

*Anal plate* simple and glabrous, with a little base, the base like a Shuriken; posterior median forked part 5.8 times as long as broadest basal part.

####### Remarks.

This species resembles *H. macropus* Scheller, 2009 from the Philippines and *H. clava* Scheller, 2013 from Australia. They can be readily distinguished by the shape of the anal plate (with little Shuriken base in *H. quadrangulus* sp. n. vs. broad base and two lateral spines in both *H. macropus* and *H. clava*) and by the numbers of the setae on tergite IV (6+6 setae in *H. quadrangulus* sp. n. and *H. clava* vs. 6+4 setae in *H. macropus*).

## Discussion

Pauropoda is a group of tiny soil myriapods, usually less than 2 mm, with unique branched antennae, having 11 (or 12) body segments and 9 (or 10 or 11) pairs of legs (Scheller 2011b). All species lack eyes and most of them also lack a tracheal system. More than 900 species grouped in 12 families have been found in the world (Qian et al. 2015). However, pauropods are still poorly known in China. Up to now, only 42 species belonging to 12 genera and 4 families have been recorded in China, as most of them were found in southeast and east China (Qian et al. 2015). This study increased our knowledge of pauropod diversity in Tibet.

As one of the most ecologically diverse landscapes on earth, the Tibetan Plateau is home to numerous rare and endangered species, and has attracted so many taxonomists to explore the biodiversity, although it is a remote area at a high altitude. However, there is only one report on the pauropods in Tibet (Zhang and Chen 1988) before our study, probably due to their small size, cryptic behavior, and the difficulties in identification.

Two of three new species reported in this study were collected from Motuo County (northern latitude 27°33' to 29°55', east longitude 93°45' to 96°05'), in the Linzhi area of southeastern Tibet. Standing 1,000 meters above the sea level on average, Motuo is surrounded by snow-capped mountains. Meanwhile, located in the lower reaches of the Yarlung Zangbo River, Motuo has a typical sub-tropical climate, warm and rainy all year round. The diversity of plants and animals in Motuo is rich in tropical species, with many endemic species (Wu et al. 2005, Zhang 2011). In this study, a similar situation in soil-dwelling pauropods was found, especially for the genus *Hemipauropus*, which is recorded for the first time in China. This genus has been found in all main zoogeographical regions, but rarely in temperate areas (Scheller 2011b). The morphology of the *Hemipauropus* species are very close to some genera of the Pauropoda distributed in the tropics.

Since our collecting sites in Tibet are still very sparse, we have not found the species of *Sphaeropauropus* sp. reported by Zhang and Chen (1988) and further investigations should be made in the future so as to reveal the diversity of Pauropoda in this area.

## Supplementary Material

XML Treatment for Decapauropus biconjugarus

XML Treatment for Decapauropus tibeticus

XML Treatment for Hemipauropus quadrangulus
